# Agar Extraction By-Products from *Gelidium sesquipedale* as a Source of Glycerol-Galactosides

**DOI:** 10.3390/molecules23123364

**Published:** 2018-12-19

**Authors:** Salim Lebbar, Mathieu Fanuel, Sophie Le Gall, Xavier Falourd, David Ropartz, Philippe Bressollier, Vincent Gloaguen, Céline Faugeron-Girard

**Affiliations:** 1 Laboratoire PEIRENE, Faculté des Sciences et Techniques, Université de Limoges, 123 Avenue Albert Thomas, 87060 Limoges CEDEX, France; salim.lebbar21@gmail.com (S.L.); philippe.bressollier@unilim.fr (P.B.); vincent.gloaguen@unilim.fr (V.G.); 2 SETEXAM Société d’ETudes et d’EXploitation des Algues et Produits Maritimes, km 7, Route de Tanger, Assam, BP 210, Kenitra 14000, Morocco; 3 INRA-UR1268 BIA–Plate-forme BIBS, 3 Impasse Yvette Cauchois—La Géraudière–BP 71627—44316 Nantes CEDEX 3, France; mathieu.fanuel@inra.fr (M.F.); sophie.le-gall@inra.fr (S.L.G.); xavier.falourd@inra.fr (X.F.); david.ropartz@inra.fr (D.R.)

**Keywords:** *Gelidium sesquipedale*, seaweed, galactosylglycerol, floridoside derivatives, (Gal)_2–4_-glycerol

## Abstract

Alkaline treatment is a common step largely used in the industrial extraction of agar, a phycocolloid obtained from red algae such as *Gelidium sesquipedale*. The subsequent residue constitutes a poorly valorized by-product. The present study aimed to identify low-molecular-weight compounds in this alkaline waste. A fractionation process was designed in order to obtain the oligosaccharidic fraction from which several glycerol-galactosides were isolated. A combination of electrospray ion (ESI)-mass spectrometry, ^1^H-NMR spectroscopy, and glycosidic linkage analyses by GC-MS allowed the identification of floridoside, corresponding to Gal-glycerol, along with oligogalactosides, i.e., (Gal)_2–4_-glycerol, among which α-d-galactopyranosyl-(1→3)-β-d-galactopyranosylα1-2–glycerol and α-d-galactopyranosyl-(1→4)-β-d-galactopyranosylα1-2–glycerol were described for the first time in red algae.

## 1. Introduction

Agar is one of the most common phycocolloids used as gelling agents in the food industry, biotechnology, and cosmetics [1]. It is extracted from red seaweeds—the so-called agarophytes. Agar is mostly extracted from algae belonging to the two genera *Gelidium* and *Gracilaria*. Agar, the major cell wall component of these algae, is a polysaccharide consisting mainly of a succession of agarobiose units, i.e., 3,6-anhydro-4-*O*-β-d-galactopyranosyl-l-galactose. Industrial agar extraction comprises several steps, generally beginning with an alkaline treatment which results in the desulfation of this polymer and, consequently, the improvement of its gelling properties [2]. Following alkaline treatment, several rinses are necessary before the solubilization of agar with hot water under pressure. This extraction process generates high amounts of by-products which are usually discarded since they are considered as waste. However, food processing by-products obtained from plants or algae are known as potential and important sources of valuable compounds [3,4].

**Figure 1:**
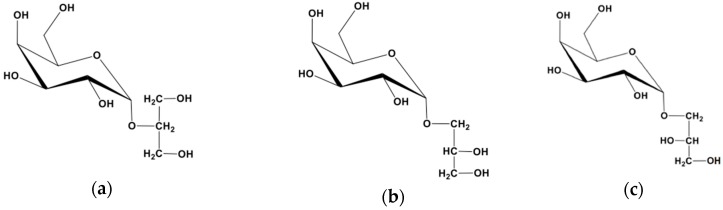
Structures of floridoside and isofloridoside: (**a**) floridoside; (**b**) d-isofloridoside; (**c**) l-isofloridoside.

Among them, low-molecular-weight carbohydrates (LMWC) such as floridoside (2-*O*-α-d-galactopyranosylglycerol) are commonly found in red algae [5], along with isofloridoside (α-d-galactopyranosyl-(1-1)-glycerol, Figure 1).

This heteroside is involved in osmoregulation [6]; its intracellular concentration is proportional to the external osmotic pressure. As one of the main products of carbon fixation during photosynthesis, floridoside is also a precursor of cell wall polysaccharides [6,7]. Furthermore, a number of biological effects have been attributed to floridoside, such as anti-inflammatory [8], antifouling [9], anti-freezing [10], neuroprotective [11], antioxidative [12], and bone growth-stimulating activities [13]. Another glycerol-galactoside, digalactosylglycerol (α-d-galactopyranosyl-(1→6)-β-d-galactopyranoside), has also been described in the red algal genus *Hypoglossum* [5,14].

Since by-products of industrial agar extraction from *Gelidium sesquipedale* are produced in high amounts and are currently poorly valorized, our research work aimed to find bioactive compounds in this waste, with a special focus on low-molecular-weight carbohydrates.

## 2. Results

**Figure 2:**
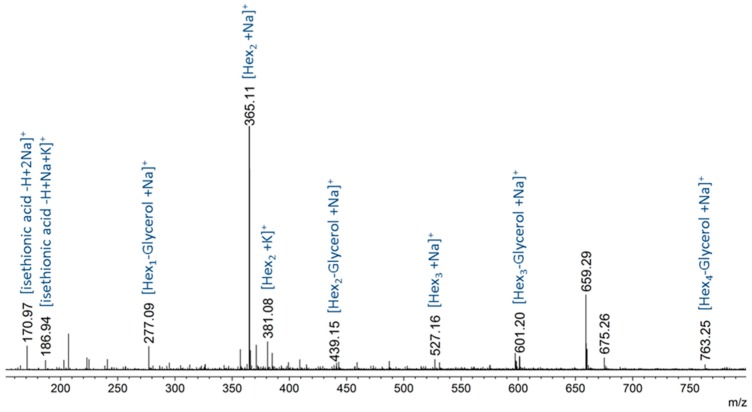
Electrospray ion (ESI)-MS (+) spectrum of the fraction containing the (hexose)_1–4_-glycerol species.

The alkaline extract of *G. sesquipedale* thalli is one of the main by-products obtained during industrial agar extraction. It was fractionated in order to recover the oligosaccharidic fraction after the elimination of polymers by ethanolic precipitation and size-exclusion chromatography of the ethanolic extract. The retained fraction, which represented 8.4% of the algal dry mass, was analyzed by electrospray ion (ESI)-MS in the positive and negative ion modes. The spectrum acquired in the positive ion mode revealed the presence of (hexose)_1–4_-glycerol (Figure 2). In addition, two peaks corresponding to isethionic acid were also detected (*m*/*z* 170.97 and *m*/*z* 186.94 as [M − H + 2Na]^+^ and [M − H + Na + K]^+^ adducts, respectively).

**Figure 3:**
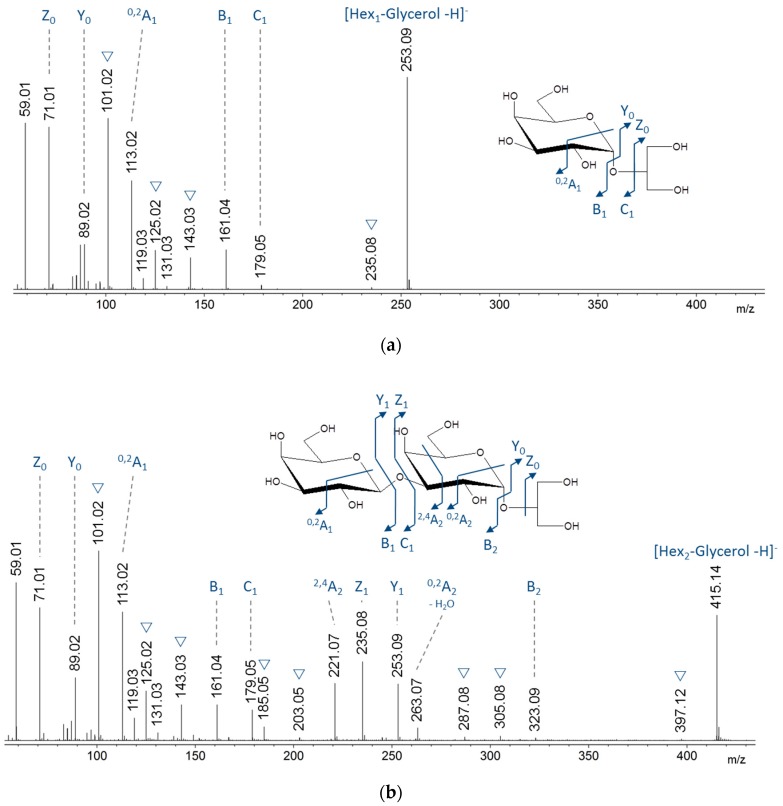
ESI-MS/MS (−) spectra of the hexose-glycerol (**a**) and the (hexose)_2_-glycerol (**b**) observed at *m*/*z* 253.09 and *m*/*z* 415.14, respectively. ▽: Water losses.

Structural characterization of species at *m*/*z* 253.09 and 415.14, attributed to hexose-glycerol and (hexose)_2_-glycerol, respectively, were performed by tandem mass spectrometry (MS/MS) in negative ionization mode. This ionization mode was selected because it produced numerous fragments of interest to confirm the structure of floridoside. The MS/MS spectrum of the *m*/*z* 253.09 species (Figure 3a) is consistent with the previously described fragmentation spectrum of floridoside [15]. The MS/MS spectrum of the *m*/*z* 415.14 species shared the same fragments originated from floridoside with an additional loss of one hexose unit from the precursor ion (Figure 3b). Regarding the linkage between the two hexoses, the intracyclic fragments ^0,2^A2 and ^2,4^A2 proved that the hydroxyl functions at positions 2 and 6 are free but they did not discriminate between positions 3 and 4.

Positive ionization mode MS/MS spectra were acquired for the following two reasons: (1) the other (hexose)*_n_*-glycerol species appeared as small peaks in negative ionization mode, and (2) the predominant [M + Cl]^−^ ion did not fragment into product ions. These experiments validated the nature of these species by comparing their fragments with those of the [M + Na]^+^ ion at *m*/*z* 439.15 of the (hexose)_2_-glycerol previously validated in negative ionization mode (comparison (hexose)_2_-glycerol/(hexose)_3_-glycerol in Supplementary Data 1). Since the predominant fragments came from glycosidic cleavages, these spectra provided no information about the type of linkages between these hexoses.

**Figure 4:**
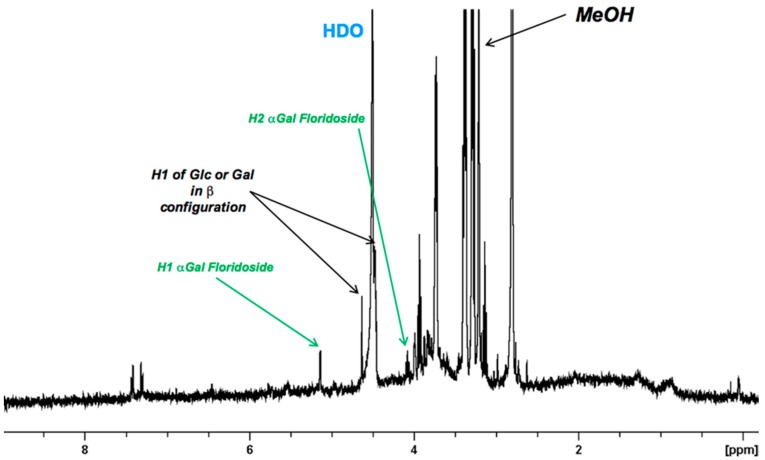
^1^H-NMR spectrum with floridoside annotations (in green).

^1^H-NMR analyses were also performed and revealed typical chemical shifts of floridoside at 5.13 ppm (proton covalently linked to the anomeric carbon of galactose) and 4.09 ppm (Figure 4), previously identified by Chen et al. [15] and Obando et al. [16]. Moreover, chemical shifts at 4.4 to 4.6 ppm suggested the presence of glucose or galactose in the β configuration; these chemical shifts are characteristic of anomeric protons of β-glucan and/or β-galactan [17,18]. We thus expected the presence of at least one galactose—or glucose—unit linked in the β configuration to the galactose residue of floridoside.

Structural analyses and glycosidic linkage analyses were also performed (Table 1) on the oligosaccharidic fraction.

As expected, galactose was mainly found in the terminal position (t-Gal, 88%/Σether) confirming the presence of floridoside; (1,3) and (1,4)-linked galactose residues were also detected and represented 6.2% and 5.7%/Σether, respectively.

**Figure 5:**
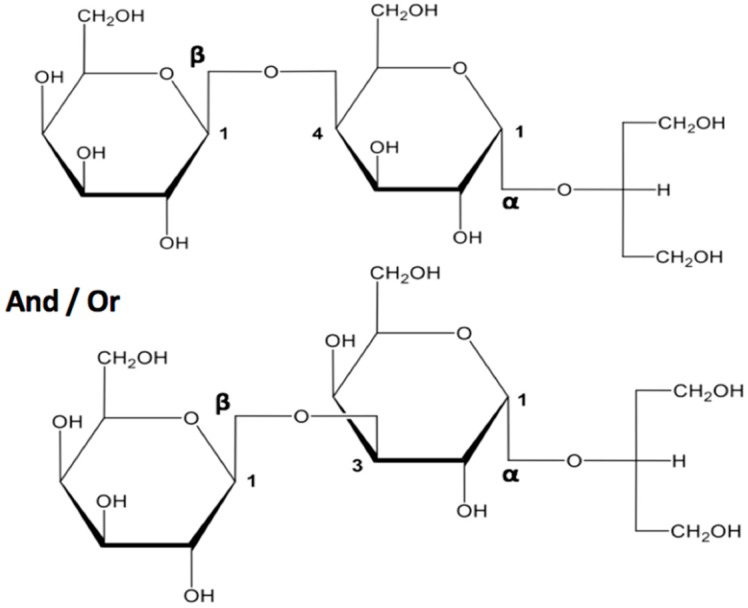
Structures of Gal_2_-Glycerol found in the alkaline extract of *Gelidium sesquipedale*.

The correlation of ^1^H-NMR and GC-MS analyses made it possible to conclude that the (hexose)*_n_*-glycerol compounds consisted of either Gal β1-3–Gal α1-2–glycerol or Gal β1-4–Gal α1-2–glycerol (Figure 5).

## 3. Discussion

The floridoside derivatives α-d-galactopyranosyl-(1→3)-β-d-galactopyranosylα1-2–glycerol and α-d-galactopyranosyl-(1→4)-β-d-galactopyranosylα1-2–glycerol are described here for the first time in *G. sesquipedale* (Table 2). (Gal)_2_-glycerol, already found in some phytoplankton species [19] and in the red algae genus *Hypoglossum* [14], consists of two galactose residues in β(1→6) linkage, contrary to the β(1→3) and β(1→4) bonds herein described. The two additional galactoside-glycerols, namely (Gal)_3_-glycerol and (Gal)_4_-glycerol, are also new compounds found in *G. sesquipedale* as well as in rhodophyceae. (Gal)_3_-glycerol has been formerly found as an ester in *Chlorella* (chlorophyceae) [20] and (Gal)_4_-glycerol has also been found as an ester (tetragalactoside-diacyl-glycerol) in oats [21]. The saponification of such compounds could occur during the alkaline treatment, thus giving rise to the oligo galactoside-glycerol molecules described herein.

This study showed that the by-product of agar extraction from *G. sesquipedale* could represent a new source of value-added molecules whose biological activities have already been described, notably floridoside [8,12] and also a mix of isethionic acid and floridoside [22]. Furthermore, we have previously shown that the oligosaccharidic fraction obtained from the alkaline treatment of *G. sesquipedale* could have elicitor activity on plants, highlighting the value-added potential of these by-products in agronomy [23]. The estimated global yield of this fraction (8.4% of the dry algal biomass) is also in agreement with the idea that the alkaline by-product of agar extraction from this seaweed could be valorized.

## 4. Materials and Methods

### 4.1. Raw Material

*G. sesquipedale* was provided by SETEXAM (Kenitra, Morocco). Thalli were harvested during the summer of 2014 on the Morocco Atlantic coast (city of El Jadida) and air-dried. However, *G. sesquipedale* thalli may carry the epiphyte *Plocamium cartilagineum*, along with impurities (corals, sand, etc.). For this reason, *G. sesquipedale* was rinsed 2 × 30 min in water at room temperature in order to eliminate the various impurities, and to make *G. sesquipedale* less rigid in order to facilitate the manual detachment of its epiphyte. Then, *G. sesquipedale* thalli were dried in an oven at 40 °C for at least 24 h.

### 4.2. Alkali Treatment

To reproduce the industrial process at a laboratory scale, 100 g of dried *G. sesquipedale* thalli were added to 1.5 L of 2% NaOH (*w*/*v*) and the mixture was warmed up to 75 °C over 1 h with gentle stirring. Afterwards, the alkaline solution was recovered by filtration through a 53-µm nylon filter. To preserve the organic compounds present in the alkaline extract, 1% H_2_SO_4_ solution was added to the filtrate until neutrality.

### 4.3. Fractionation of the Alkaline Extract

After neutralization, the alkaline extract was concentrated by rotary evaporation (Heidolf, Schwabach, Deutschland) under vacuum and three volumes of ethanol were added. After homogenization, the mixture was left overnight at 4 °C, and the precipitate was then separated from the ethanolic fraction by centrifugation (2000× *g*, 20 min). The ethanolic extract was concentrated by rotary evaporation and subjected to size exclusion chromatography on Bio-Gel P2 (2.5 × 70 cm, Bio-Rad, Hercules, CA, USA, fractionation range: 100–1800 Da) with demineralized water as the eluent. Each deposit corresponded to the equivalent of 0.2 g of ethanolic extract in 10 mL ultrapure water. After elution, orcinol-positive fractions were collected and kept for further analysis.

### 4.4. Electrospray Mass Spectrometry Analyses

Experiments were performed on a Synapt G2Si high-definition mass spectrometer (Waters Corp., Manchester, UK) equipped with an electrospray ion (ESI) source. Two types of mass measurements were performed on the sample: first, a mass profile was done on a mass range of 150–2000 *m*/*z* (MS). Ions of interest were further isolated and fragmented by collision-induced dissociation in the transfer cell of the instrument (MS/MS). In the MS/MS experiments, ion mobility (IM) was activated to reduce interference from sample impurities. IM was performed in a traveling-wave ion mobility (TWIM) cell. The gas flows were held at 180 mL·min^−1^ He in the helium cell and at 90 mL·min^−1^ N_2_ in the mobility cell. The IM traveling wave height was set to 40 V, and its wave velocity was set to 550 m·s^−1^. The sample was prepared at 25 µg·mL^−1^ in MeOH/H_2_O (1:1, *v*/*v*) and then infused at a flow rate of 5 μL·min^−1^ in the instrument. The instrument was operated in positive and negative polarity, as well as in ‘sensitivity’ mode.

### 4.5. ^1^H-Nuclear Magnetic Resonance (NMR) Experiments

The sample was dissolved in 750 µL of D_2_O in a 5-mm NMR tube. NMR analyses were carried out with a Bruker Avance III 400 NB spectrometer (Bruker, Germany) using a BBo 5-mm H/X probe. The proton NMR spectrum was recorded with presaturation of the water signal.

### 4.6. Determination of the Glycosidic Linkage

The oligosaccharide fraction was dissolved in dimethylsulfoxide (DMSO, 1 mL). Oligosaccharides were then methylated, hydrolyzed, and the monomers converted into their alditol acetate derivatives before analysis with a Thermo Scientific Trace GC-ISQ mass spectrometer (Waltham, MA, USA) as described in Reference [24]. The identification of partially methylated alditol acetates was based on their retention times and combined with confirmed by mass spectra fragmentation and compared to a homemade library. Permethylation was performed in duplicate.

## 5. Conclusions

The industrial agar extraction from red seaweed generates important volumes of by-products which are poorly valorized. This study showed that the alkaline extract of *G. sesquipedale* contains LMWC, all of which belong to the glycerol-galactoside family. The smallest one, floridoside, has already been described in red algae, but a series of Gal_2–4_-glycerol harboring β1→3 and/or β1→4 linkages between galactose residues were also found and constituted original derivatives of galactosyl-glycerol in algae or plant kingdoms.

## Figures and Tables

**Table 1:** Glycosidic linkage analyses of the oligosaccharidic fraction. Values are expressed as mole percentages of the total neutral sugar derivatives identified. Gal: Galactose. t: terminal.

Linked Sugar	% Total Sugar (Mean)	Standard Deviation
t-Gal	88.0	2.6
(1,3)-Gal	6.2	0.7
(1,4)-Gal	5.7	1.9

**Table 2:** Low-molecular-weight compounds identified in the oligosaccharidic fraction of the alkaline extract of *G. sesquipedale*.

Compounds	Molecular Weight
Isethionic acid	126
3,6-Anhydrogalactose	162
Hexose	180
Floridoside (Gal-glycerol)	254
Disaccharide (hexose)	342
Gal_2_-glycerol	416
Trisaccharide (hexose)	504
Gal_3_-glycerol	578
Disaccharide (hexose)	666
Gal_4_-glycerol	740
Yield (algal dry mass)	8.4%

## Supplementary Materials

The Supplementary Materials are available online.
