# Supplementary material for: Agar Extraction By-Products from Gelidium sesquipedale as a Source of Glycerol-Galactosides
Source: Molecules. 2018 Dec 19;23(12):3364. doi: 10.3390/molecules23123364 (PMC6320990; doi:10.3390/molecules23123364)
Supplement: Supplementary file 1 [file molecules-23-03364-s001.pdf]

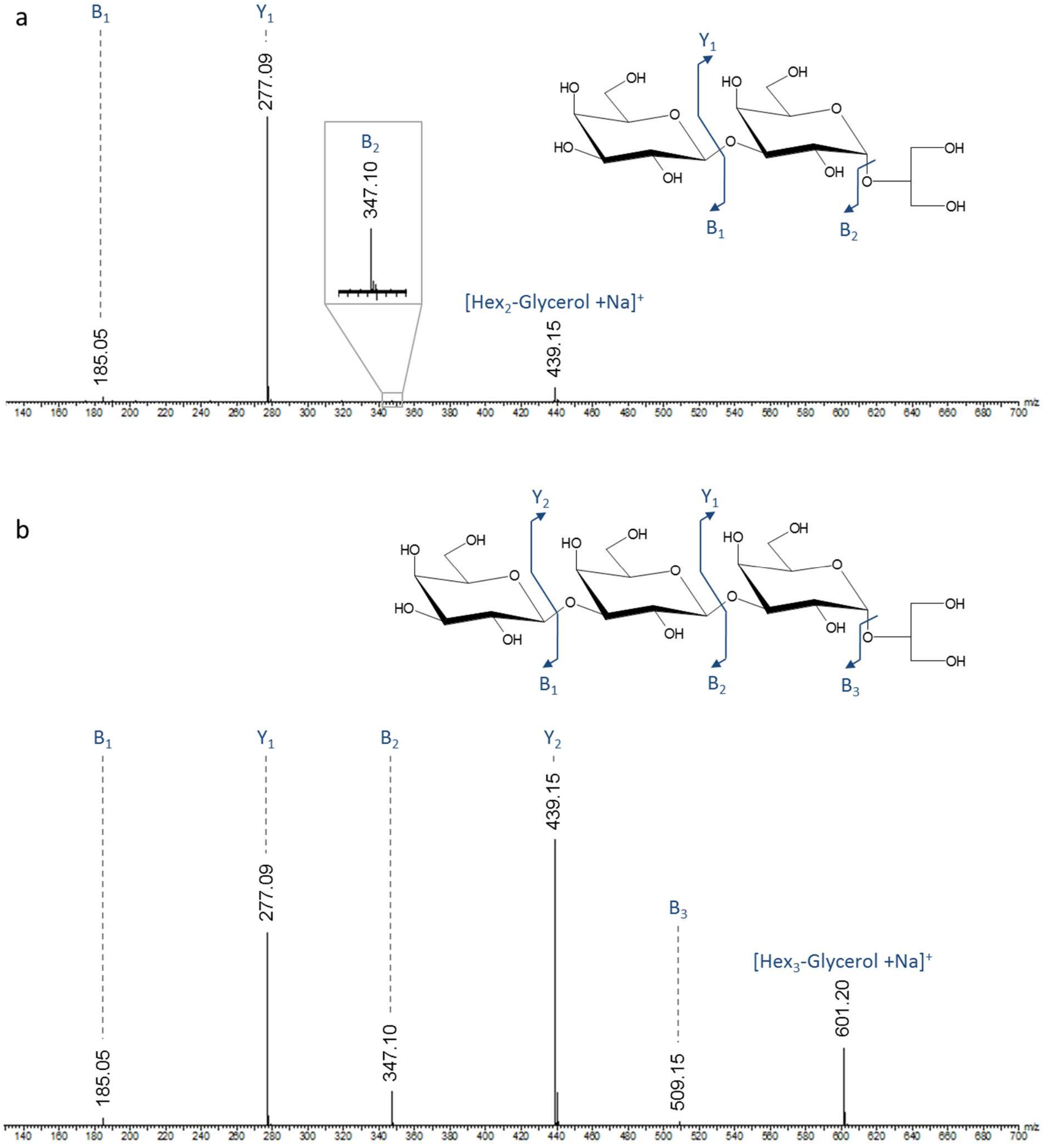

Supplementary data 1. ESI-MS/MS (+) spectra of the (hexose)2-glycerol (**a**) and the (hexose)3-glycerol (**b**) observed at *m*/*z* 439.15 and *m*/*z* 601.20 respectively.
